# Safety of Recombinant Thrombomodulin for Severe Gastroenterological Sepsis-Induced Coagulopathy in Patients Undergoing Invasive Digestive Procedures: A Single-Center Retrospective Cohort Study

**DOI:** 10.7759/cureus.93379

**Published:** 2025-09-27

**Authors:** Takahisa Fujikawa, Keiji Nagata

**Affiliations:** 1 Surgery, Kokura Memorial Hospital, Kitakyushu, JPN

**Keywords:** digestive disease, disseminated intravascular coagulation (dic), invasive procedure, recombinant human soluble thrombomodulin, sepsis

## Abstract

Introduction

Several studies demonstrated that anticoagulant treatment, including the administration of recombinant human soluble thrombomodulin (rTM), could improve the outcomes of patients with gastroenterological sepsis-induced disseminated intravascular coagulation (DIC). However, there is little evidence on the safety of rTM administration for septic DIC requiring invasive procedures (IPs). In the current study, we assessed the safety and efficacy of rTM in patients with sepsis-induced DIC associated with digestive diseases.

Methods

The current retrospective cohort study included 155 cases in which rTM was administered for septic DIC associated with digestive diseases at our hospital between 2014 and 2023. The subjects were divided into an IP group (n = 102) and a non-IP group (n = 53), and background factors, the presence or absence of rTM-related complications, and mortality were compared between the groups.

Results

CHADS_2_ and CHA_2_DS_2_-VASc scores were similar between the groups, although the Sequential Organ Failure Assessment (SOFA) score was significantly higher in the IP group than in the non-IP group (5 vs. 3, p = 0.008). The IP group had significantly more patients taking oral antithrombotic (61% (62/102) vs 43% (23/53), p = 0.043) and more cases at high risk of bleeding with a HAS-BLED score of 3 or higher (70% (71/102) vs 43% (25/53), p = 0.009) than the non-IP group. The rTM was administered for a median of six days, and the DIC scores significantly improved from 5.5 to 2.9 points after rTM administration (p < 0.0001). The rTM-related bleeding complications were observed in nine cases (5.8%), and the overall survival rate was 78.7% (122/155), with no significant difference between the groups. Multivariate analysis revealed that the factors influencing mortality were the DIC score after rTM administration (p = 0.006) and performance status (p = 0.017), but not the presence or absence of IPs.

Conclusion

Septic DIC associated with digestive system diseases is a pathological condition with a poor prognosis, but rTM administration improved DIC without increasing bleeding complications, regardless of the presence or absence of IPs. Administration of rTM might be safe and does not increase bleeding complications, even in high-risk patients requiring IPs.

## Introduction

Excessive coagulation and vascular endothelial damage, which result in the production of intravascular thrombin and fibrin, are the hallmarks of disseminated intravascular coagulation (DIC). As a result, small- to medium-sized arteries develop thrombosis, which leads to serious bleeding and organ failure [1]. In sepsis, the expression of thrombomodulin, an anticoagulant in the vascular endothelium, is inhibited by inflammatory cytokines, and subsequent excessive production of thrombin results in the formation of multiple microthrombi, which exacerbates sepsis with DIC [2]. Severe sepsis continues to result in a high mortality rate, with estimates averaging between 20% and 30% [3]. Reports indicate that DIC worsens 20%-40% of sepsis cases [4,5], with a significantly elevated death rate among affected patients [6]. These factors have led to its status as a significant public health issue, with an estimated annual cost of over $20 billion to the United States' healthcare system [7].

The prognosis for patients with sepsis and DIC is exceedingly poor, because multiple organ failure can develop, making early diagnosis and intervention crucial in the treatment of sepsis. For sepsis-induced DIC, the anticoagulant substance recombinant human soluble thrombomodulin (rTM) has been developed. Bleeding complications have been reported as adverse incidents of rTM therapy, especially in patients requiring invasive procedures (IPs), such as endoscopic biliary drainage or emergency laparotomy [8]. Theoretically, the anticoagulant effect of rTM is attenuated as the thrombin level decreases, which reduces the incidence of bleeding complications [9]. Still, few studies have focused on the risk of bleeding complications during rTM treatment in patients with gastroenterological sepsis-induced DIC who require IPs.

In the current study, we assessed the safety of rTM in patients with sepsis-induced DIC associated with digestive diseases, with special reference to those who required IPs.

## Materials and methods

**Figure 1:**
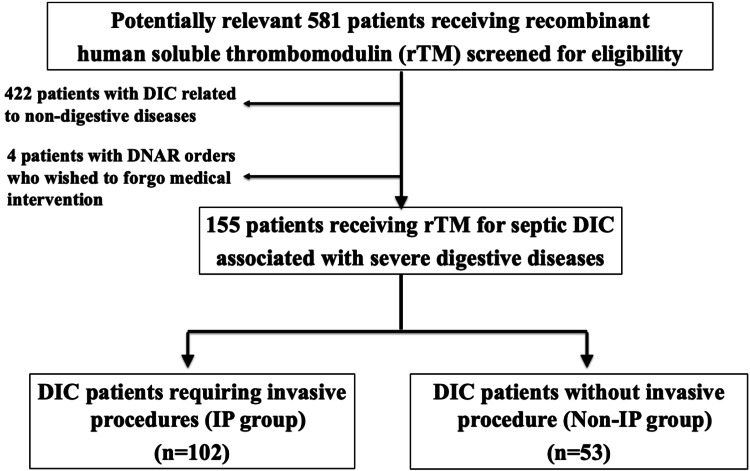
Consort diagram in the current study rTM, recombinant human soluble thrombomodulin; DIC, disseminated intravascular coagulation; IP, invasive procedure

In the current retrospective cohort study, we received approval from the Institutional Review Board at Kokura Memorial Hospital, Kitakyushu, Japan, and the single institution's prospectively gathered DIC database was reviewed for potentially pertinent cases. After excluding cases of other types of DIC, those related to non-digestive diseases, and patients with DNAR (Do-Not-Attempt Resuscitation) orders who wished to forgo medical intervention, we included a total of 155 consecutive septic DIC patients with acute digestive inflammatory diseases from January 2014 to December 2023 in the current study (Figure 1). To analyze the background and surgical factors of the whole cohort, the patients were divided into two groups according to the presence or absence of IPs: 102 patients who required IPs for acute inflammatory disease (IP group), and 53 patients with septic DIC without IPs (non-IP group).

The criteria for administering rTM (Asahi Kasei Pharma Corp., Tokyo, Japan) were met when DIC was diagnosed. Patients with DIC were diagnosed as having a Japanese Association for Acute Medicine (JAAM) DIC score of 4 or more [10]. The typical dosage of rTM was 380 U/kg/day for a duration of six days. The dosage of rTM was reduced to 130 U/kg/day in instances of severe renal impairment (CKD stage G4 or G5). All patients received treatment based on the judgment of the attending physicians, with no restrictions on the concurrent use of additional anticoagulants or medications for underlying conditions and complications. Antithrombin (AT) level was generally checked at the time of DIC diagnosis, and AT concentrate was provided to patients if their level fell below 70%. In the IP group, we performed endoscopic or surgical IPs immediately (within a few hours) after diagnosis, and at the same time, started septic DIC treatment at an early stage.

The patients’ symptoms and functional status were reported according to the Eastern Cooperative Oncology Group Scale of Performance Status (PS) [11]. The Sequential Organ Failure Assessment (SOFA) score was used to assess the patient's severity. The HAS-BLED score was utilized to assess bleeding risk, with a score of 3 or above indicating clinical importance [12]. To assess the potential thromboembolic risk for patients, we adopted the revised CHADS_2_ and CHA_2_DS_2_-VASc scoring systems [13,14]. Both scoring systems define patients as being categorized into low- (score 0), moderate- (score 1), and high-risk groups (score 2 or higher). Patients with DIC were identified using the JAAM DIC diagnostic criteria (DIC score ≥ 4) [10]. Changes in DIC scores were measured on the first day of rTM administration and on days 5-7.

The primary outcomes included mortality and rTM-related bleeding complications, including luminal bleeding (e.g., gastrointestinal bleeding), abdominal bleeding, and abdominal wall hematoma. The background characteristics, perioperative factors, and surgical outcomes of the included patients were compared between the groups, and the independent risk factors for patient mortality were determined by multivariate analyses.

Statistical analysis 

Continuous values were expressed as mean (standard deviation) or median (range), while categorical variables were presented as absolute numbers and percentages. For univariate comparisons, Fisher’s exact test was used to evaluate categorical variables; alternatively, continuous variables were analyzed using Student’s t-test or the Mann-Whitney U test for normally or non-normally distributed data, respectively. Multivariate logistic regression analysis was performed to determine risk factors affecting overall mortality. All p-values were two-sided, and values less than 0.05 were considered statistically significant. All statistical analyses were performed using EZR (Saitama Medical Center, Jichi Medical University, Saitama, Japan), which is a graphical user interface for R version 2.13.0 (The R Foundation for Statistical Computing, Vienna, Austria) [15].

## Results

Patient and operative characteristics

**Figure 2:**
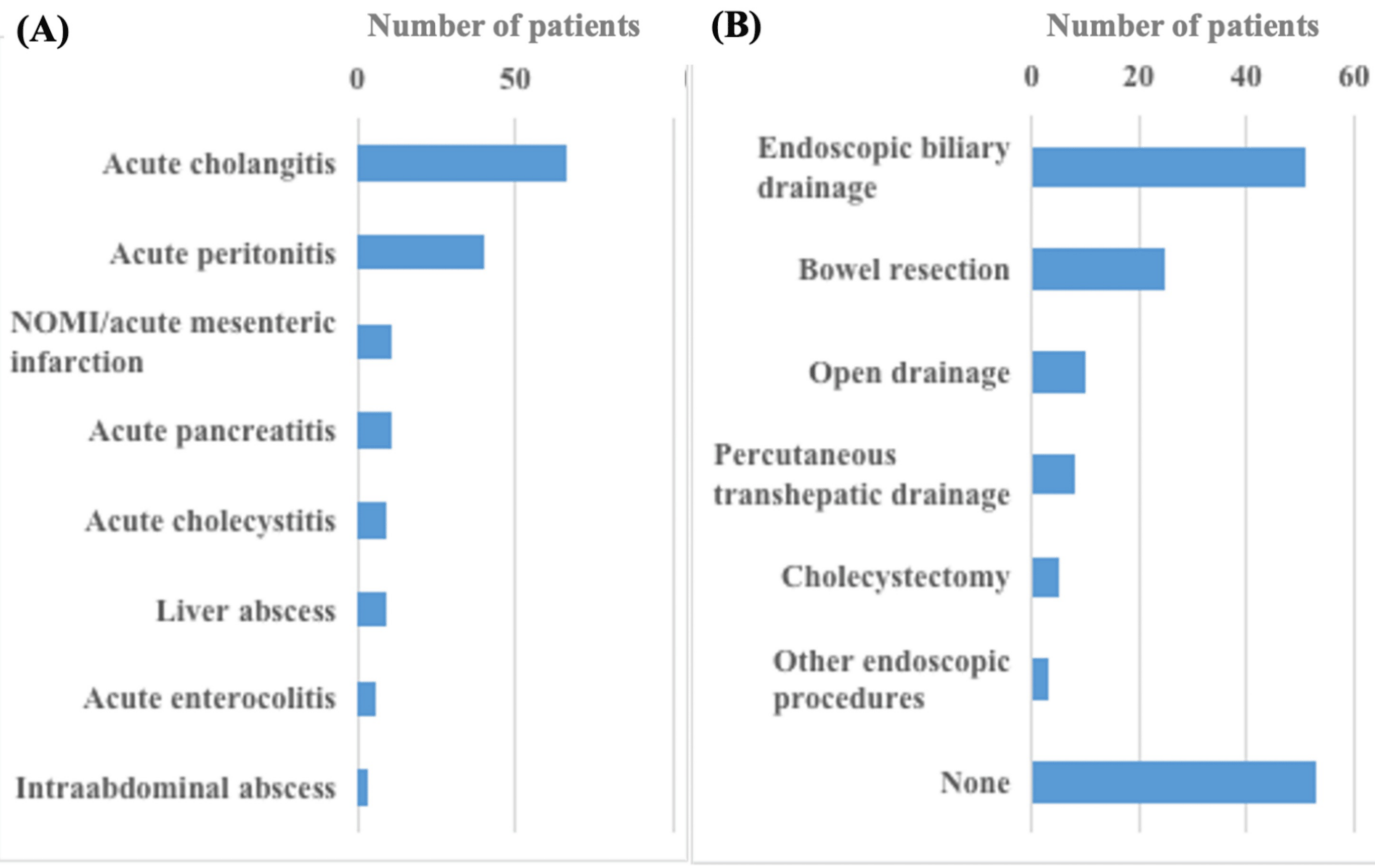
Underlying disease that caused DIC and the types of invasive procedures in the current cohort (A) Concerning the underlying disease, acute cholangitis and acute peritonitis were the major indications for the administration of rTM. (B) As for the types of invasive procedures, endoscopic biliary drainage was dominant, and bowel resection and open drainage followed. NOMI, non-obstructive mesenteric ischemia; DIC, disseminated intravascular coagulation; rTM, recombinant human soluble thrombomodulin

The number of patients in the IP and non-IP groups was 102 (65.8%) and 53 (34.2%), respectively (Figure 1). Figure 2A shows the types of underlying diseases that cause DIC. In the current cohort, acute cholangitis and acute peritonitis were the major indications for the administration of rTM, accounting for 42.6% and 25.8% of the whole cohort, respectively. Figure 2B shows the types of IPs in the cohort. Endoscopic biliary drainage was dominant, with a rate of 32.9%, followed by bowel resection (16.1%) and open drainage (6.5%).

Table 1 shows the patient characteristics in each group. The median ages in the non-IP and IP groups were 77 years (range, 36 to 93 years) and 76 years (range, 44 to 94 years), respectively (p = 0.257). Patients with male gender (p = 0.034), poor PS (status 2-4, p = 0.040), hypertension (p = 0.008), a history of congestive heart failure (p = 0.007), maintenance of peritoneal dialysis or hemodialysis (p = 0.031), and the use of oral antithrombotic therapy (p = 0.043) were more prevalent in the IP group. Regarding patient severity, the median SOFA score was significantly higher in the IP group than in the non-IP group (5 vs. 3, p = 0.008). Patients categorized as high-risk according to the revised CHADS_2_ scoring system comprised 64.2% and 79.4% of the non-IP and IP groups, respectively (p = 0.053). Additionally, those categorized as high-risk according to the CHA_2_DS_2_-VASc score comprised 88.7% and 91.2%, respectively (p = 0.775). Of the entire cohort, 54% were at high risk of bleeding, with a HAS-BLED score of 3 or higher, and a high HAS-BLED score was more prevalent in the IP group than in the non-IP group (69.6% vs. 47.2%, p = 0.009).

**Table 1:** Background characteristics of patients in the whole cohort Fisher’s exact probability test or the Chi-square test was used to evaluate categorical variables; alternatively, continuous variables were analyzed using Student’s t-test or the Mann-Whitney U test for normally or non-normally distributed data, respectively. Bold values indicate statistical significance; # values indicate t-values; and * values indicate Chi-square values. IP, invasive procedure; BMI, body mass index; Hx, history; TIA, transient ischemic attack; SOFA, sequential organ failure assessment;

Variables	Non-IP (n = 53)	IP (n = 102)	p-value	Chi-square or t-values
Age, y, median (range)	77 (36-93)	77 (44-94)	0.257	0.592#
Male gender, n (%)	28 (52.8)	72 (70.6)	0.034	4.060*
BMI, kg/m^2^, median (range)	21.5 (14.0-30.3)	22.0 (13.9-40.7)	0.217	1.574#
Performance status >=2, n (%)	4 (7.5)	21 (20.6)	0.040	3.474*
Hypertension, n (%)	31 (58.5)	81 (79.4)	0.008	6.608*
Diabetes mellitus, n (%)	14 (26.4)	35 (34.3)	0.365	0.674*
Hx of congestive heart failure, n (%)	10 (18.9)	41 (40.2)	0.007	6.253*
Hx of cerebral infarction/TIA, n (%)	7 (13.2)	23 (22.5)	0.201	1.397*
Current hemo-/peritoneal dialysis, n (%)	5 (9.4)	25 (24.5)	0.031	4.159*
Oral antithrombotic therapy, n (%)	23 (43.4)	62 (60.8)	0.043	0.112*
SOFA score, median (range)	3 (0-10)	5 (0-13)	0.008	2.992#
Revised CHADS_2_ score >=2, n (%)	34 (64.2)	81 (79.4)	0.053	3.483*
CHA_2_DS_2_-VASc score >=2, n (%)	47 (88.7)	93 (91.2)	0.775	0.045*
HAS-BLED score >=3, n (%)	25 (47.2)	71 (69.6)	0.009	6.527*

Safety and efficacy of rTM administration

**Figure 3:**
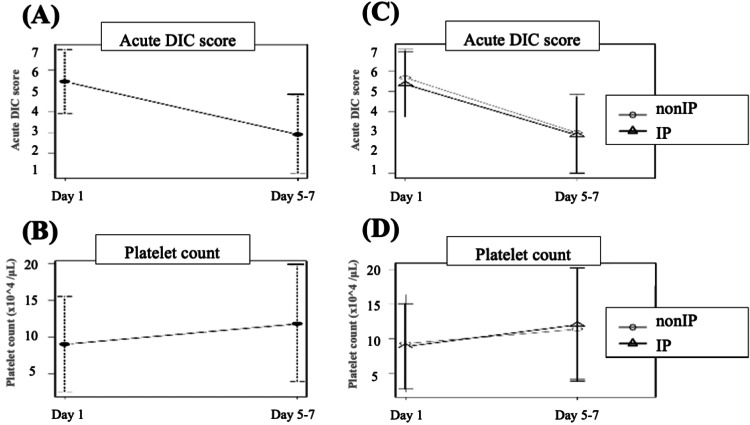
Changes in the JAAM DIC score and platelet count after the initiation of rTM therapy (A, B) Changes of the DIC score (A) and platelet count (B) in the whole cohort. (C, D) Comparisons of changes of the DIC score (C) and platelet count (D) between the non-IP and IP groups. The markers and bars are represented as the mean value ± standard error. JAAM, Japanese Association for Acute Medicine; DIC, disseminated intravascular coagulation; IP, invasive procedure; rTM, recombinant human soluble thrombomodulin

Table 2 shows the factors regarding rTM administration and therapeutic outcomes in each group. In the IP group, the numbers of endoscopic and surgical treatments were 54 (34.8%) and 43 (27.7%), respectively. The administration of rTM lasted for a median of six days. Figure 3 shows the changes in the JAAM DIC score and platelet count after the initiation of rTM therapy. Concerning the efficacy of rTM administration for septic DIC, both the JAAM DIC score (p < 0.001) and platelet count (p < 0.001) were significantly ameliorated from the first day to days 5-7 of rTM administration (Figures 3A-3B). In terms of IPs, there were no significant differences in the changes in JAAM DIC score and platelet count between the groups (Figures 3C-3D).

**Table 2:** Factors regarding rTM administration and therapeutic outcomes in each group Fisher’s exact probability test or the Chi-square test was used to evaluate categorical variables; alternatively, continuous variables were analyzed using Student’s t-test or the Mann-Whitney U test for normally or non-normally distributed data, respectively. # values indicate t-values; and * values indicate Chi-square values. rTM, recombinant human soluble thrombomodulin; IP, invasive procedure; DIC, disseminated intravascular coagulation; AT-III, antithrombin III; RBC, red blood cell; NA, not available

Variables	Non-IP (n = 53)	IP (n = 102)	p-value	Chi-square or t-values
Type of invasive treatment: Endoscopic, n (%)	NA	54 (34.8)	NA	NA
Type of invasive treatment: Surgical, n (%)	NA	43 (27.7)	NA	NA
Platelet count (the day of rTM initiation), x10^4^ /μL, median (range)	7.0 (1.7-30.4)	7.9 (1.5-47.5)	0.591	0.388#
Platelet count (5-7 days after rTM initiation), x10^4^ /μL, median (range)	9.6 (2.4-36.4)	10.0 (2.6-46.5)	0.817	0.472#
DIC score (the day of rTM initiation), median (range)	5 (3-8)	5 (3-8)	0.298	1.223#
DIC score (5-7 days after rTM initiation), median (range)	3 (0-7)	3 (0-7)	0.604	0.373#
Duration of rTM therapy, days, median (range)	6 (2-14)	6 (2-10)	0.707	0.437#
Use of AT-III, n (%)	11 (20.8)	29 (28.4)	0.338	0.710*
Use of heparin, n (%)	2 (3.8)	8 (7.8)	0.496	0.402*
RBC transfusion during rTM therapy, unit, median (range)	0 (0-8)	0 (0-20)	0.952	0.505#
Length of post-therapeutic stay, days, median (range)	24 (5-107)	23 (6-149)	0.530	1.290#
rTM-related bleeding complication, n (%)	2 (3.8)	7 (6.9)	0.719	0.175*
Mortality, n (%)	12 (22.6)	21 (20.6)	0.837	0.008*

Concerning the therapeutic outcomes, rTM-related bleeding complications were observed in nine cases (5.8%), and the overall mortality rate was 21.3%, with no significant difference between the two groups. In the non-IP group, two rTM-related bleeding events occurred (intra-abdominal bleeding in one, and bleeding from a gastric ulcer in one), both of which occurred during the late phase of rTM administration and were conservatively treated. In the IP group, three gastrointestinal bleedings, two intra-abdominal bleedings, one nasal bleeding, and one biliary hematoma occurred during the late phase of rTM administration, all of which were surgically or endoscopically treated.

Risk factors affecting the overall mortality of septic DIC patients

Univariate and multivariate analyses for overall mortality in the whole cohort were conducted to elucidate the risk factors. In the univariate analysis, a history of chronic heart failure (p = 0.038), a history of cerebral infarction or transient ischemic attack (p = 0.044), poor PS (grade 2 or higher, p = 0.001), a high DIC score after rTM administration (score 4 or higher, p = 0.002), and a high HAS-BLED score (score 3 or higher, p = 0.048) were associated with overall mortality. 

**Figure 4:**
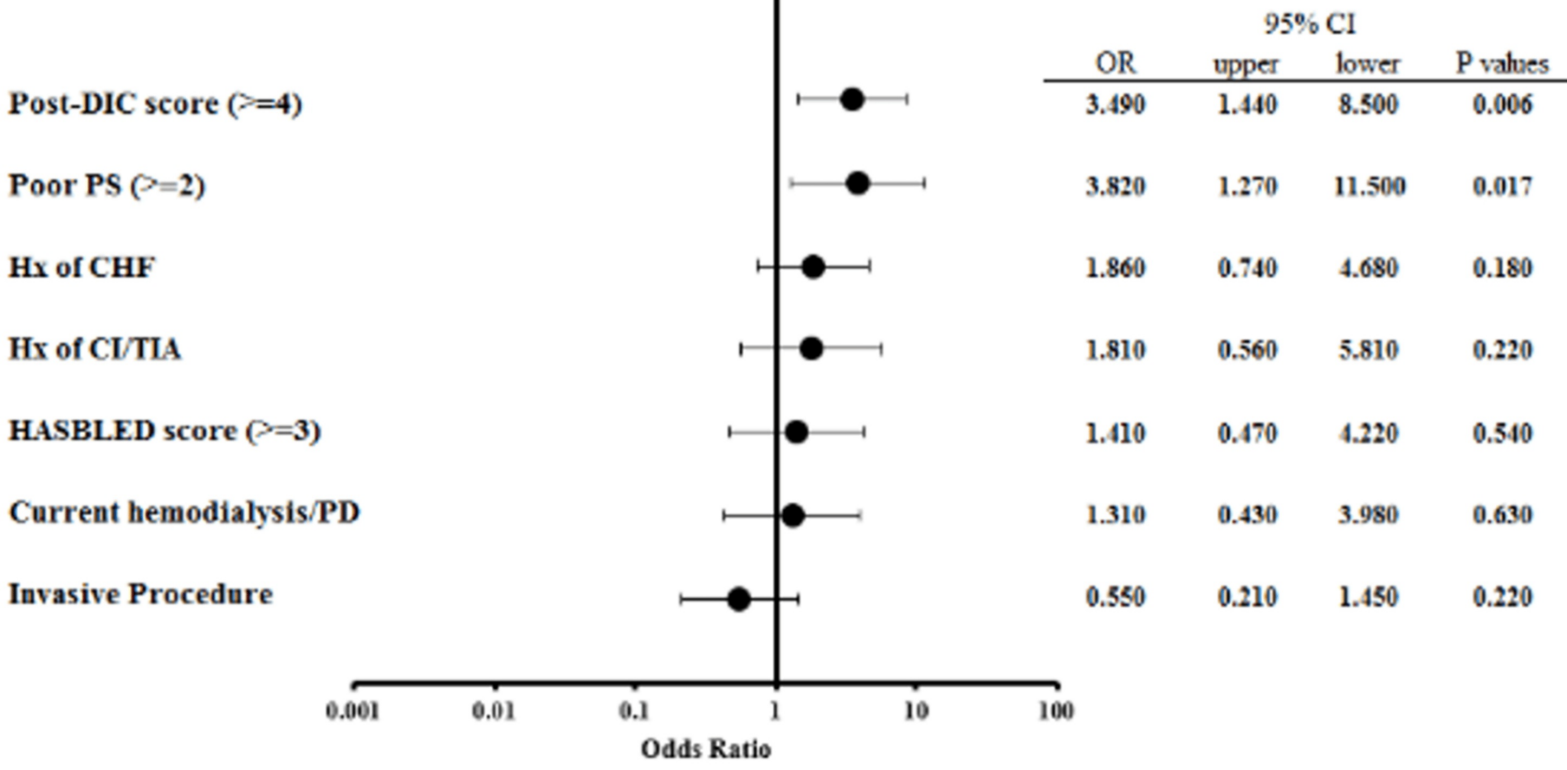
Forest plots showing odds ratios of overall mortality in the whole cohort Multivariate logistic regression analysis was performed to determine risk factors that affect overall mortality. DIC, disseminated intravascular coagulation; PS, performance status; CHF, chronic heart failure; CI, cerebral infarction; TIA, transient ischemic attack; PD, peritoneal dialysis; 95% CI, 95% confidence interval; OR, odds ratio.

To elucidate the effect of IPs on overall mortality, we conducted a multivariate logistic regression analysis in the whole cohort. Figure 4 shows the results of the multivariate analysis for overall mortality. In the multivariate analysis, the factors influencing mortality were persistently high DIC scores (≥4) after rTM administration (p = 0.006) and poor PS (p = 0.017), but not the presence or absence of IPs (p = 0.220).

## Discussion

The current retrospective cohort study assessed 155 cases in which rTM was administered for septic DIC associated with digestive diseases. All patients received rTM for a median of six days, which significantly improved their DIC scores, and the overall survival rate was 78.7%. In the IP group, 61% were taking oral antithrombotic drugs, and 70% were at high risk of bleeding. rTM-related bleeding complications were observed in nine cases (5.8%), with no significant difference between those with or without IPs. Multivariate analysis revealed that the factors influencing mortality were the DIC score after rTM administration (p = 0.006) and performance status (p = 0.017), but not the presence or absence of IPs. Our results suggest that rTM administration may be safe and effective in treating septic DIC associated with digestive diseases, even when IPs are required.

Anticoagulant substances such as rTM have been developed and are currently widely used for the management of septic DIC. Thrombomodulin connects with thrombin, forming a thrombin-thrombomodulin-α complex that activates protein C to its active state, which then inactivates factors VIIIa and Va, in conjunction with protein S, thereby preventing additional thrombin production [16]. The efficacy of rTM in sepsis-induced DIC has also been documented based on its anti-inflammatory properties via the lectin-like domain, leading to the inhibition of lipopolysaccharide [17,18].

From the clinical perspective, two meta-analyses and one phase III randomized controlled study indicated the efficacy of rTM in sepsis-associated coagulopathy [16,19,20], but one randomized study (SCARLET trial) indicated no decrease in mortality with the administration of rTM for septic DIC [21]. Despite the ongoing debate over the clinical utility of rTM for the treatment of septic DIC, some encouraging research findings about septic DIC linked to digestive disorders, such as acute cholangitis or coagulopathy following gastrointestinal, hepatobiliary, or pancreatic surgery, have been reported [22-24]. Accordingly, the Tokyo Guideline 18 for acute cholangitis-induced DIC showed that the administration of rTM may be considered for severe cholangitis complicated with DIC [25].

Little evidence exists regarding the safety of rTM use for treating septic DIC, particularly in patients who require invasive gastroenterological procedures. Despite the attenuation of rTM's anticoagulant action with decreasing thrombin levels, which mitigates the frequency of bleeding issues, such complications remain a concern as an adverse event associated with rTM therapy. One study regarding rTM use in 32 patients with sepsis-induced DIC following emergency abdominal surgery for gastrointestinal necrosis or perforation reported that no perioperative bleeding complications were observed and that rTM was safely used even in critically ill patients with high bleeding risks [22]. The current study assessed 155 patients with gastroenterological sepsis-induced DIC, including those at high risk of bleeding requiring IPs. This study showed that rTM-related bleeding complications were observed in 5.8% of patients, all of which occurred in the late phase of rTM administration and were successfully managed endoscopically or surgically. In addition, the rate of bleeding complications was not significantly different between those with and without IPs. These results suggest that, even when IPs are required in patients with septic DIC associated with acute digestive diseases, rTM may be safely administered without an increase in the occurrence of bleeding complications.

Limitations

It has a few limitations. Initially, the present investigation is a retrospective observational study conducted at a single center. Although multivariate analysis was employed to account for baseline differences between the groups, it is feasible that unmeasured covariates were not accounted for, which could have led to residual treatment selection bias. Secondly, there is heterogeneity in IPs (ranging from endoscopic drainage to major surgery) and the potential difficulty in attributing bleeding events solely to rTM, which may affect the generalizability of the findings. Last but not least, our institution is a tertiary referral hospital that treats a high volume of surgical patients with high bleeding risks, including those receiving antithrombotic therapy. Consequently, our results may not be applicable to centers with a lower volume of patients. Multi-institutional investigations will alleviate this restriction.

## Conclusions

The prognosis for septic DIC, a pathological condition associated with digestive system maladies, is poor. However, the administration of rTM improved DIC without increasing bleeding complications, irrespective of the presence or absence of IPs. Administration of rTM might be safe and does not increase bleeding complications, even in high-risk patients requiring IPs.
